# Analysis of accuracy of four types of fully guided dental implant surgical guides: an in vivo study

**DOI:** 10.1186/s12903-026-08969-1

**Published:** 2026-06-24

**Authors:** Abdelrahman K. Eldabe, Doaa Adel-Khattab, George N. Farid Beshay, Kirollos H. Botros

**Affiliations:** 1 Periodontology and Diagnosis, Faculty of Dentistry, Assiut University, Assiut, Egypt; 2 Oral Medicine, Periodontology and Oral Diagnosis, Faculty of Dentistry, Ain Shams University, Cairo, Egypt; 3 Periodontology and Diagnosis, Faculty of Dentistry, Assiut University, Assiut, Egypt

**Keywords:** Fully guided surgical guides, Stackable guides, Mucosa supported guides, Unilateral tooth supported guides, Bilateral tooth supported guides

## Abstract

**Objective:**

This clinical trial was conducted to compare the four types of static fully guided surgical guides (bilateral tooth-supported guides, unilateral tooth-supported guides, mucosa-supported complete arch guides, and stackable bony-supported guides) in terms of accuracy.

**Aim:**

To compare all static guided protocols (bilateral tooth supported, unilateral tooth supported, full arch mucosa supported, stackable bone supported) with each other under homogenous conditions, accuracy is defined as the closeness of spatial agreement between any given implant as planned (reference) and as inserted (measurement), expressed by four spatial deviation parameters.

**Methods:**

All patients in this investigation underwent a cone beam computer tomography with standard settings. Meanwhile, digital impressions were taken by an intraoral scanner. Implant planning software was used, considering a prosthetic-driven approach. Fully guided surgeries were executed. To evaluate the four groups of fully guided surgical guides, a digital imprint was obtained immediately after surgery. All STL files were imported into CAD software, and a digital library translated scan bodies to implant fixture replicas. For trueness tests, new STL files were loaded into an inspection program.

**Results:**

A single experienced surgeon inserted 86 implants using four different types of fully guided surgical guides. For the angular deviation, the Kruskal-Wallis test showed that there was no significant difference (*P*=.117). In contrast, for the coronal global and apical global deviations, as well as the vertical discrepancies, there was a statistically significant difference between the four groups (*P*=.022), (*P*=.018), (*P* < .001), respectively. A Dunn-Bonferroni test was used to compare the groups in pairs to identify any significant differences.

**Conclusions:**

Given the limitations of this study, the trueness of the four categories of fully guided surgical guides falls within an acceptable range. Nevertheless, when utilizing mucosa-supported and stackable guides, operators should consider inherent deviations and exercise heightened vigilance.

**Clinical Trial Registry Date:**

27. 04. 2025.

**Clinical Trial Registry Number:**

NCT06947057.

## Background

The rising prevalence of older age groups globally has contributed to an increased requirement for dental implant surgical procedures, underscoring the importance of enhancing surgical precision. This improvement directly enhances success rates and minimizes surgical trauma. As a means of transmitting information on implant direction, position, and angle, digital surgical guides can significantly improve surgical accuracy while cutting down on operating time and difficulties [1]. The methods of data collecting, manufacturing processes, supporting types, fixation screws, and surgical guide sleeve design all affect the surgical precision of dental implantation [2].

Surgical guides have been developed to limit positional imprecision encountered during freehand surgical procedures. These guides are categorized into dynamic and static types. Although they allow continuous intraoperative imaging, dynamic systems are less accurate than static ones [3]. They also cost a lot of money, take up a lot of space, and require specialized expertise to operate [4]. Static systems work with templates, and in most clinical scenarios, their accuracy is sufficient. These templates are primarily created through 3D printing using digital images from CBCT/intraoral scanners. After aligning DICOM files from CBCT with STL files from scanning oral tissues, either directly through intraoral scanners or indirectly through extraoral scanners, the resulting template is supported by either bone, mucosa, or teeth [5].

The degree of guidance determines three distinct protocols that apply to the use of the templates. The initial drill, or “pilot drill surgical guide,” in which the pilot drill step is only guided, and the resulting bone socket is meant to serve as a guide for later osteotomies and implant placement. Only implant placement is done without the template in the partially (or half-) guided technique. All osteotomies are done using the template. The partial guidance technique offers the most guidance possible for any implant method without a completely guided kit. Finally, the fully guided protocol, where the guide is utilized in both ostomy site creation and implant placement [6].

Surgical guides may be categorized according to their mode of support as tooth-supported, mucosa-supported, bone-supported, or as combinations of these support types. Tooth-supported templates are further subdivided into unilateral and bilateral designs. Bilateral tooth-supported guides are stabilized by teeth on both sides of the arch, whereas unilateral tooth-supported guides rely on teeth on one side in conjunction with mucosal or bony support on the contralateral side. Mucosa-supported guides are typically indicated for full-arch cases in which bone reduction is not required [7, 8]. When bone reduction is required, stackable guides are considered the optimal approach because they facilitate the planning of implant osteotomies and bone sculpturing using a single template consisting of multiple components [9].

Guide accuracy varies according to the type of support employed, with bilateral tooth-supported guides theoretically achieving greater retention and biomechanical stability through anchorage on hard tissues. A recent systematic review reported that unilateral tooth-supported guides demonstrated greater overall deviations, except for global coronal deviation, in which unilateral designs exhibited marginally higher accuracy than bilateral guides. Mucosa-supported guides were associated with the lowest in vivo accuracy, while their in vitro performance was considered less reliable due to pronounced discrepancies between experimental models and human mucosal tissues [8]. Current evidence regarding the validity of stackable guides is limited; nevertheless, reported preliminary outcomes fall within acceptable parameters [9–11].

Even with the increasing predictability of guided surgery, discrepancies between the virtual plan and actual clinical execution remain unavoidable [1]. The accuracy of static computer-aided implant surgery was evaluated in the 2018 consensus document issued by the International Team for Implantology. The reported mean deviations were 1.2 mm at the crestal point, 1.4 mm at the apical point, 3.5°, 0.2 mm for coronal depth, and 0.5 mm for apical depth. Based on the previously reported accuracy values, a safety margin of 2 mm should always be considered [5].

To our knowledge, no prior clinical trials have compared all static guided protocols (bilateral tooth supported, unilateral tooth supported, full arch mucosa supported, stackable bone supported) with each other under homogenous conditions. For the purpose of this study, accuracy is defined as the degree of spatial agreement between the planned implant position (reference) and the inserted implant position (measurement), as quantified by four spatial deviation parameters.

## Methods

### Research question

**Fig. 1:**
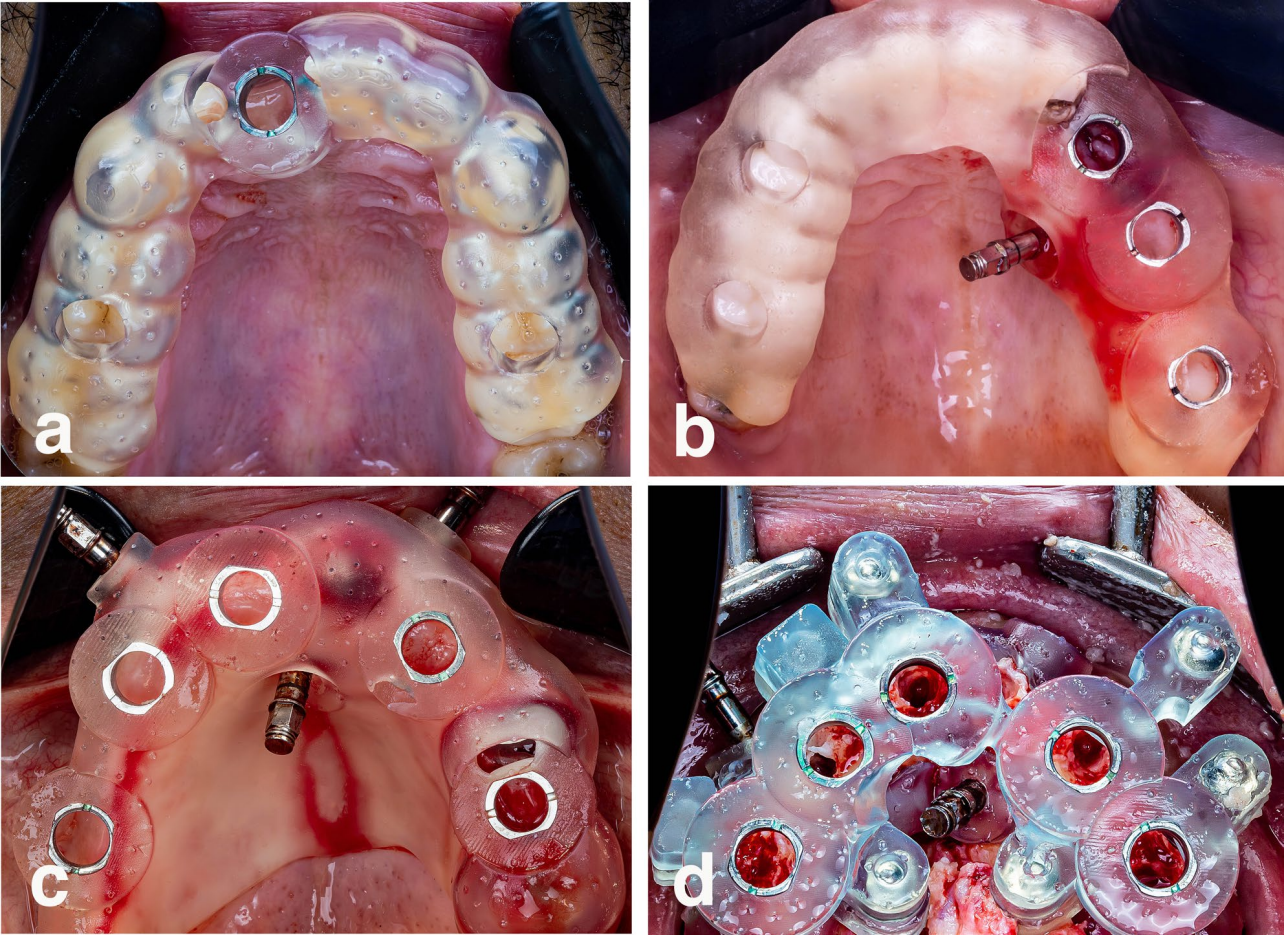
The four types of fully guided surgical guides: (**a**) bilateral tooth-supported guides, (**b**) unilateral tooth-supported guides, (**c**) mucosa-supported complete arch guides, and (**d**) stackable bone-supported guides. To ensure stability and retention, fixation pins were utilized in all surgical guides except the bilateral tooth-supported guides

This study was performed following the PICO approach (Patient, Intervention, Comparison, and Outcome). The PICO question was formulated as follows:

In patients requiring dental implants, do the four types of fully guided surgical guides (bilateral tooth-supported guides, unilateral tooth-supported guides, mucosa-supported complete arch guides, and stackable guides) result in the same degree of accuracy (trueness) (Fig. 1)?

- Population: Patients seeking dental implants
- Intervention: Use of fully guided surgical guides (bilateral tooth-supported guides, unilateral tooth-supported guides, mucosa-supported complete arch guides, and stackable guides)
- Comparison: Comparison between the four types of fully guided surgical guides
- Outcome: Trueness (accuracy) of implant placement

The null hypothesis was that the four types of fully guided surgical guides would show equivalent accuracy (Degree of trueness).

### Study design and registration

All participants were recruited from the outpatient clinic of the Oral Medicine, Periodontology, and Oral Diagnosis Department, Faculty of Dentistry, Ain Shams University, between May 2025 and October 2025. Before enrollment, patients were informed that their data would be used for academic purposes, and written informed consent was obtained. Ethical approval for the study was granted by the Ain Shams Institutional Ethical Committee, affiliated with the Egyptian Network of Research Ethics Committees (Address: Faculty of Dentistry, Ain Shams University; FDASU-Rec IR032423). All patients were treated in accordance with regional laws, good clinical practice, and in adherence to the Declaration of Helsinki (1996). In addition, the study was registered in the clinical trial registration site www.clinicaltrials.gov on 27. 04.2025, (NCT06947057).

### Recruitment of participants

The study was conducted at the Faculty of Dentistry, Ain Shams University, Egypt, and participants were recruited from the university outpatient clinic. Inclusion criteria comprised adults aged 25 to 65 years who were missing a single tooth or were partially or fully edentulous, and whose clinical condition was considered suitable for implant placement by the examining physician or principal investigator, based on satisfactory soft- and hard-tissue conditions and occlusion. Exclusion criteria included the presence of systemic conditions that could compromise healing or bone metabolism (such as uncontrolled diabetes or hyperthyroidism), a history of radiotherapy, chemotherapy, or bisphosphonate therapy, pregnancy or planned pregnancy during the study period, and smoking.

### Outcomes

The primary outcome measure was angular deviation (AD), defined as the angle, expressed in degrees, formed between the principal axis of the planned implant and that of the inserted implant. Secondary outcome measures included coronal global deviation (CGD), defined as the distance between the centers of the coronal ends of the planned and inserted implants; apical global deviation (AGD), defined as the distance between the apical endpoints of the planned and inserted implants; and vertical deviation, defined as the vertical linear distance between the centers of the coronal ends of the planned and inserted implants.

### Sample size calculation

A power analysis was performed to ensure sufficient statistical power to test the null hypothesis that accuracy did not differ among the tested groups. The analysis was conducted using an alpha (α) level of 0.05, a beta (β) level of 0.20 (corresponding to a statistical power of 90%), and an effect size (Cohen’s f) of 0.481, derived from the results of a previously published study [6]. The required total sample size was calculated to be 52 implants, corresponding to 13 implants per group. Implant location was considered the unit of analysis. Sample size estimation was carried out using R statistical software (version 4.3.2 for Windows).

### Blinding

Blinding of the surgeon was not feasible. Outcome assessors could not be blinded. The biostatistician was blinded to the current clinical trial.

### Virtual implant planning and surgical guide fabrication

Cone beam computed tomography (CBCT) scans were obtained using the Veraview X800 L P system (JMorita Mfg. Corp., Kyoto, Japan) with identical standard settings for all participants (100 kV, 8 mA, 9 s, voxel size: 250 μm, field of view: 110 mm). All scans included in the study were acquired using these parameters. In parallel, digital impressions were obtained with the Aoral Scan3 intraoral scanner (Shining 3D, China). The digital impressions accurately captured the surface topography of the dental arches, including gingival tissues, teeth, and existing prosthetic structures. These high-resolution surface data were essential for restoration design and prosthetic outcome planning.

Subsequently, the acquired CBCT datasets and digital impression files were imported into the Exoplan software version Galway 3.0 (Exocad; Darmstadt, Germany). The software supports multiple file formats, including DICOM for CBCT data and STL for digital impressions. Initial dataset alignment was performed either by manual selection of corresponding anatomical landmarks on both datasets and, in edentulous cases, by applying the dual-scan technique. Intelligent software algorithms facilitated the alignment of these reference points to establish an initial dataset match. Following this step, advanced registration algorithms within Exoplan were used to refine the alignment through a fine-tuning process. This process employed iterative closest point (ICP) algorithms to minimize discrepancies between the CBCT and digital impression datasets. Continuous evaluation and refinement of the alignment ensured accurate superimposition of the digital impression surface topology onto the corresponding regions of the CBCT scan. This step was essential to achieve high precision, as even minor misalignments may adversely affect surgical guide accuracy and implant placement [12].

The prosthetically driven planning approach implemented in the Exoplan software enabled simultaneous consideration of surgical and prosthetic factors, thereby ensuring that implant positioning corresponded with the intended restorative outcomes. Based on the finalized treatment plan and the aligned datasets, fully guided surgical guides were subsequently designed. These fully guided guides controlled the precise trajectory of the surgical drills and implants, effectively translating the digital plan into accurate clinical execution and ensuring adherence to the planned implant positions. All cases were planned by a single experienced clinician (K.H.B.) and independently verified by the operating surgeon (A.K.). The surgical guides were fabricated using biocompatible materials with a DLP 3D printer (AccuFab D1s; Shining 3D, China).

### Presurgical procedures

All participants underwent ultrasonic scaling before the scheduled surgical procedure. Participants were instructed on the importance of maintaining adequate oral hygiene. In addition, a prophylactic antibiotic regimen was implemented, consisting of amoxicillin/clavulanic acid (2,000 mg, orally) or clindamycin (600 mg, orally) administered 60 min before surgery.

### Surgical procedures

**Fig. 2:**
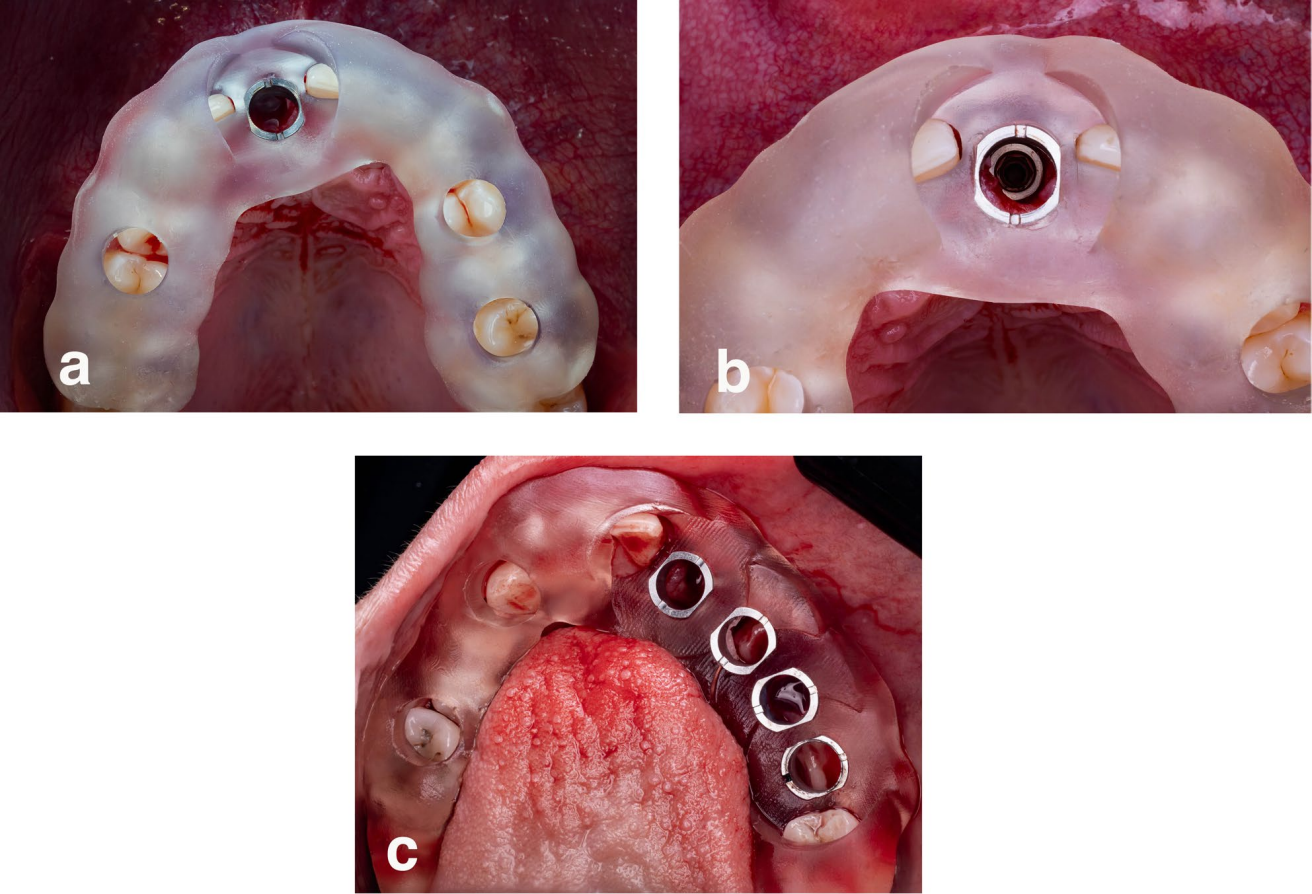
Cases of bilateral tooth-supported guides. The stability and retention of the guide are gained from the neighboring teeth on both sides. **a**-**b **Case with a single tooth gap. **c **case with multiple tooth gaps. Full seating of the guide is evaluated through the windows

**Fig. 3:**
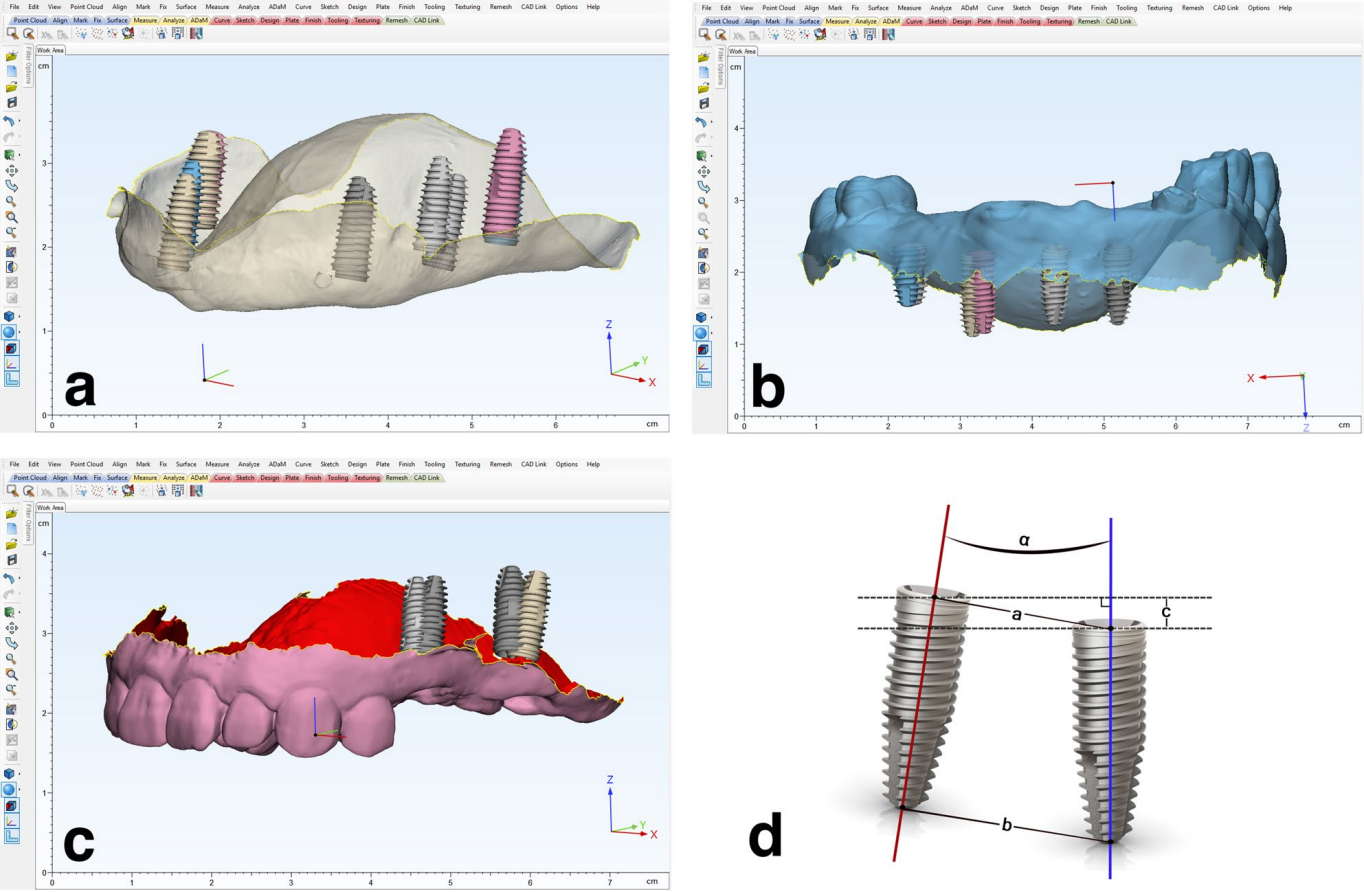
**a**, **b **Cases of unilateral tooth-supported guide. To ensure stability and retention, fixation pins were utilized. **a **fixation through the implant (**b**) fixation through the bone. **c**-**d **Cases of mucosa-supported guide. Fixation by multiple fixation pins is preferred to ensure stability throughout the whole procedure

**Fig. 4:**
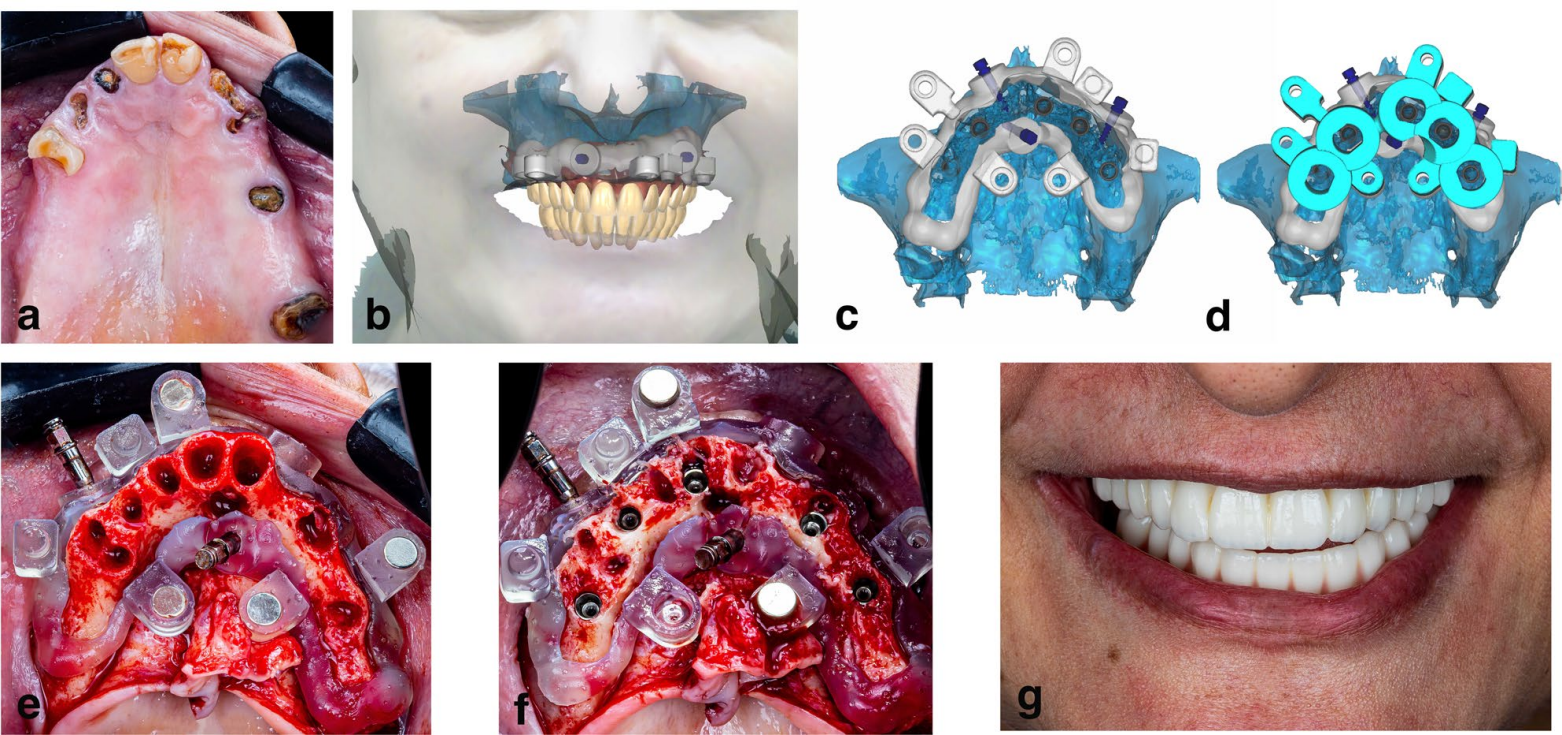
**a**-**f **Case of a stackable guide with a scalloping bone reduction guide for bone recontouring before final fixture placement through the implant placement guide. **g** Monolithic screw retained zirconia prosthesis in the patient’s mouth

After administration of local anesthetics (articaine with adrenaline 1:100.000), volunteers were instructed to rinse with 0.2% chlorhexidine solution for 30 s. All surgeries were performed by one experienced surgeon (A.K.). The template was placed and checked for complete seating through the inspection windows created in it, and both the osteotomies and fixture insertion were performed through the guide (Fig. 2). The guided surgical kit, NEO NAVIGUIDE kit (Neobiotech, Korea), was used, and fully guided surgeries were executed following the manufacturer’s instructions. All implants were Neobiotech IO IS II active of various dimensions. Fixation pins were utilized in all surgical guides except the bilateral tooth-supported guides. Fixation pins ensured stability and retention of the guide throughout the surgical procedure (Fig. 3). After 3 months, all volunteers received screw-retained monolithic zirconia restorations (Fig. 4). Patients were checked clinically and radiographically at 6 and 12 months after surgery.

### Postoperative care

All patients received postoperative medications for one week, including amoxicillin/clavulanic acid (1 g twice daily for 7 days; Augmentin, GlaxoSmithKline, Egypt) and metronidazole (500 mg three times daily for 7 days; Flagyl, Sanofi Aventis, Egypt). An anti-inflammatory agent was prescribed three times daily for 7 days (Alphintern, Amoun, Egypt), and ibuprofen (600 mg; Brufen, Kahira Pharmaceuticals, Egypt) was administered when severe pain was reported.

Patients were instructed to rinse twice daily with a 0.12% chlorhexidine digluconate mouthwash (Antiseptol; Kahira Pharm, Egypt) and to avoid mechanical plaque removal at the implant site for 15–30 days. A soft diet was recommended during the initial postoperative period. No provisional or definitive prosthesis exerting pressure on the implants was used throughout the entire implant healing phase.

### Data processing and trueness assessment

Immediately following surgery, a digital impression was obtained using scan bodies as fixture locators to evaluate the accuracy of the fully guided surgical guides across the four study groups. All scans were acquired by the same experienced operator using an intraoral photogrammetry scanner (Elite, Shining 3D, China) and compatible scan bodies. To guarantee the highest degree of accuracy possible for fixture position capturing, especially for full-arch scans, intraoral photogrammetry was used [13, 14]. A standardized scanning protocol was followed, a continuous zigzag pattern from occlusal, buccal and lingual surfaces. Patients were seated upright and cheek retractors (Optragate, Ivoclar Vivadent, Switzerland) were used to improve visibility and moisture control. The resulting STL files were imported into dental CAD software (Exocad DentalCAD 3.1 Rijeka; Exocad, Darmstadt, Germany), where scan bodies were converted into implant fixture replicas using a digital library, thereby generating a model that represented the actual implant position. The updated STL files were subsequently imported into inspection software (Mimics, version 2.4; Leuven, Belgium) for trueness assessment.

The postoperative STL file was superimposed onto the corresponding preoperative STL file used for planning and surgical guide fabrication in Exoplan software. Superimposition was performed using a two-step process:

1. N-point registration: The operator manually selected 3 stable reference points on the buccal surfaces of adjacent teeth and/or identifiable soft tissue landmarks (e.g., rugae area) visible in both the preoperative and postoperative scans. These points were used to initially align the two files.
2. Global registration: The software then performed an automatic fine registration using an iterative closest point (ICP) algorithm. The ICP process minimized the distance between the two surfaces by optimizing the alignment transformation. The number of iterations was set to 20 so that the probability of error is negligible.

Finally, the resulting registered scans were visually inspected in multiple views (frontal, occlusal, sagittal) to verify accurate alignment. If any discrepancies were detected, the registration process was repeated.

**Fig. 5:**
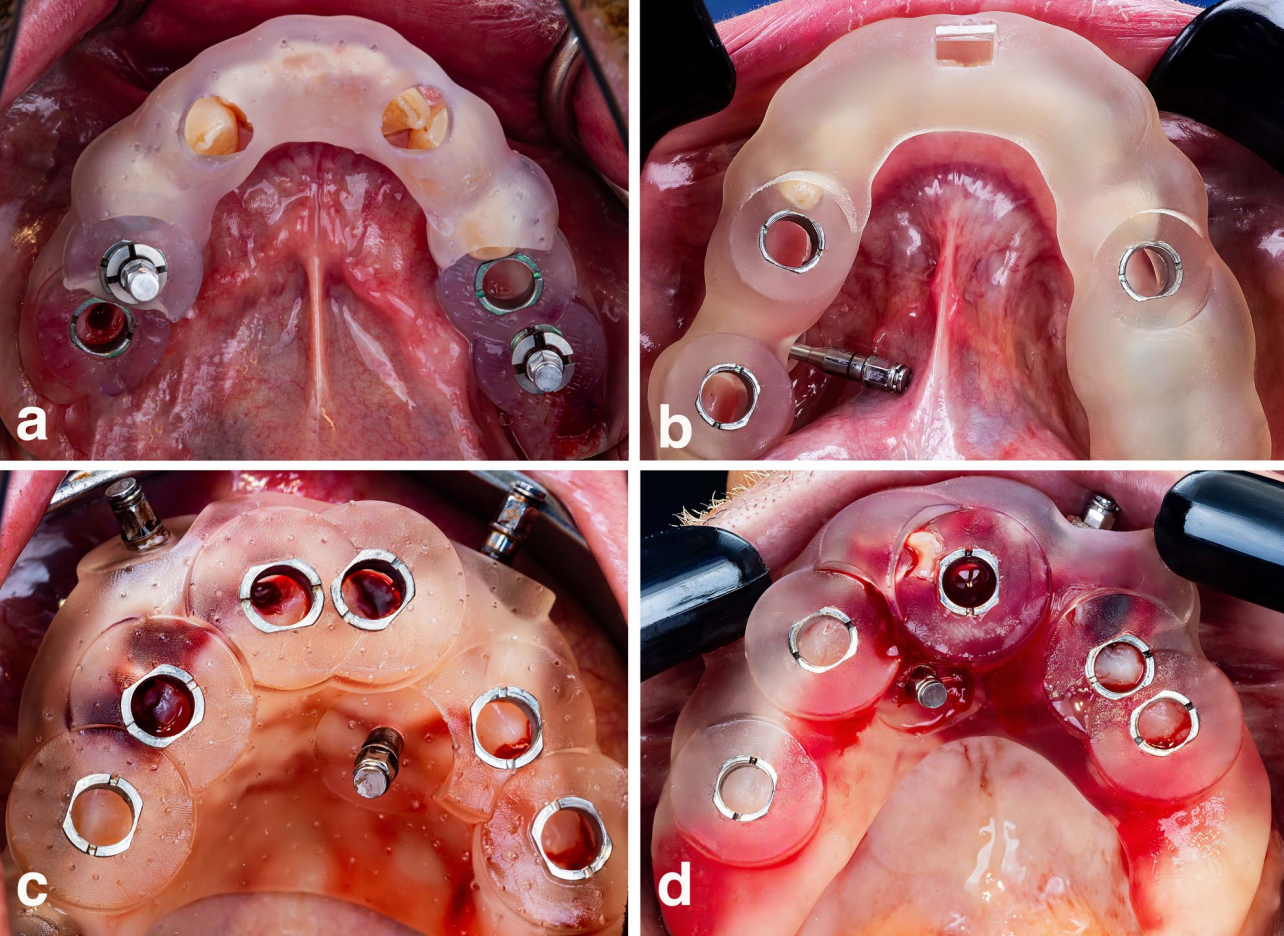
**a**-**c **STL files were imported into inspection software Mimics for trueness assessments. **d **Describes the four spatial deviations: α = angular deviations (AD) defined as the angle, expressed in degrees, formed between the principal axis of the planned implant and that of the inserted implant, a=coronal global deviation (CGD) defined as the distance between the centers of the coronal ends of the planned and inserted implants, b=apical global deviation (AGD) defined as the distance between the apical endpoints of the planned and inserted implants, and c= vertical deviation defined as the vertical linear distance between the centers of the coronal ends of the planned and inserted implants

To measure the deviations between the planned and actual implant positions, a central point and the central long axis of both the planned and inserted implant fixture replicas were identified. Then the software automatically measures the four outcomes: angular deviation (AD), coronal global deviation (CGD), apical global deviation (AGD), and vertical deviation. The coronal and apical global deviations, as well as the vertical deviation, were measured relative to the implant axis, while the angular deviation represented the angle between the two axes (Fig. 5).

### Statistical analysis

Continuous data were presented as mean and standard deviation (SD) values. Since several cases had more than one implant, measurements were averaged at the patient level to ensure statistical independence and to avoid within-subject clustering effects. The data were analyzed for normality and variance homogeneity by viewing the distribution and using the Shapiro-Wilk and Levene’s tests, respectively. They were all found to be normally distributed; however, the homogeneity assumption was violated for vertical deviation data. They were analyzed using Welch-one-way ANOVA followed by Games-Howell post hoc test. Other data were analyzed using one-way ANOVA followed by Tukey’s post hoc test. Different models were confirmed for residuals’ normality using the methods mentioned earlier. The significance level was set at *p* < .05 within all tests. Statistical analysis was performed with R statistical analysis software version 4.5.1 for Windows.

## Results

Thirty-five volunteers with indications for 92 implants were enrolled in the study. Four patients were excluded before or during the study according to the predefined exclusion criteria. Of the 31 patients finally included in the study, 10 patients were treated with bilateral tooth-supported guides (*n* = 24), 14 patients were treated with unilateral tooth-supported guides (*n* = 23), 4 patients were treated with mucosa-supported guides (*n* = 20), and 3 patients were treated with stackable guides (*n* = 19). A single experienced surgeon inserted 86 implants using four different types of fully guided surgical guides. No complications or implant losses were reported during the follow-up period. Consequently, the current survival rate stands at 100%.

Table 1 summarizes the results for angular, coronal global, and apical global deviations, as well as vertical discrepancies, to enable comparisons of planned and placed implant positions based on the type of fully guided surgical guide used.

**Table 1:** Demonstrates the results for angular, coronal global, and apical global deviations, as well as vertical discrepancies between the four types of fully guided surgical guides

Measurement	Mean ± SD				F-value	*p*-value	ω^2^ (95% CI)
	B	U	M	S			
Angular deviation	4.35 ± 1.47^A^	4.89 ± 2.42^A^	5.96 ± 1.61^A^	5.42 ± 1.16^A^	**0.70**	**0.559**	**0.00 (0.00 to 0.00)**
Coronal deviation	1.32 ± 0.54^A^	1.42 ± 0.71^A^	1.99 ± 0.62^A^	1.70 ± 0.28^A^	**1.28**	**0.301**	**0.03 (0.00 to 0.13)**
Apical deviation	1.22 ± 0.30^A^	1.59 ± 0.58^A^	1.89 ± 0.56^A^	1.72 ± 0.34^A^	**2.30**	**0.100**	**0.11 (0.00 to 0.31)**
Vertical deviation	0.04 ± 0.03^B^	0.09 ± 0.05^B^	0.20 ± 0.13^A^	0.26 ± 0.06^A^	**14.84**	< 0.001*	**0.57 (0.27 to 0.72)**

Values with different superscripts within the same horizontal row are significantly different

*CI* Confidence interval

B=Bilateral tooth-supported guide, U=Unilateral tooth-supported guide, M=Mucosa supported guide, S=Stackable guide

* significant (*p* < .05)

A Games-Howell post hoc test was used to compare the groups in pairs to find out which was significantly different. For the AD, CGD, and AGD, the test showed that the pairwise group comparisons were not significantly different from each other. For the vertical deviation, a significant difference was found between the four groups, with the bilateral tooth-supported guides revealing the least vertical discrepancy (0.04 ± 0.03 mm) while the stackable guides were associated with the highest vertical deviation (0.26 ± 0.06 mm). Thus, with the available data, the null hypothesis was partially rejected.

**Fig. 6:**
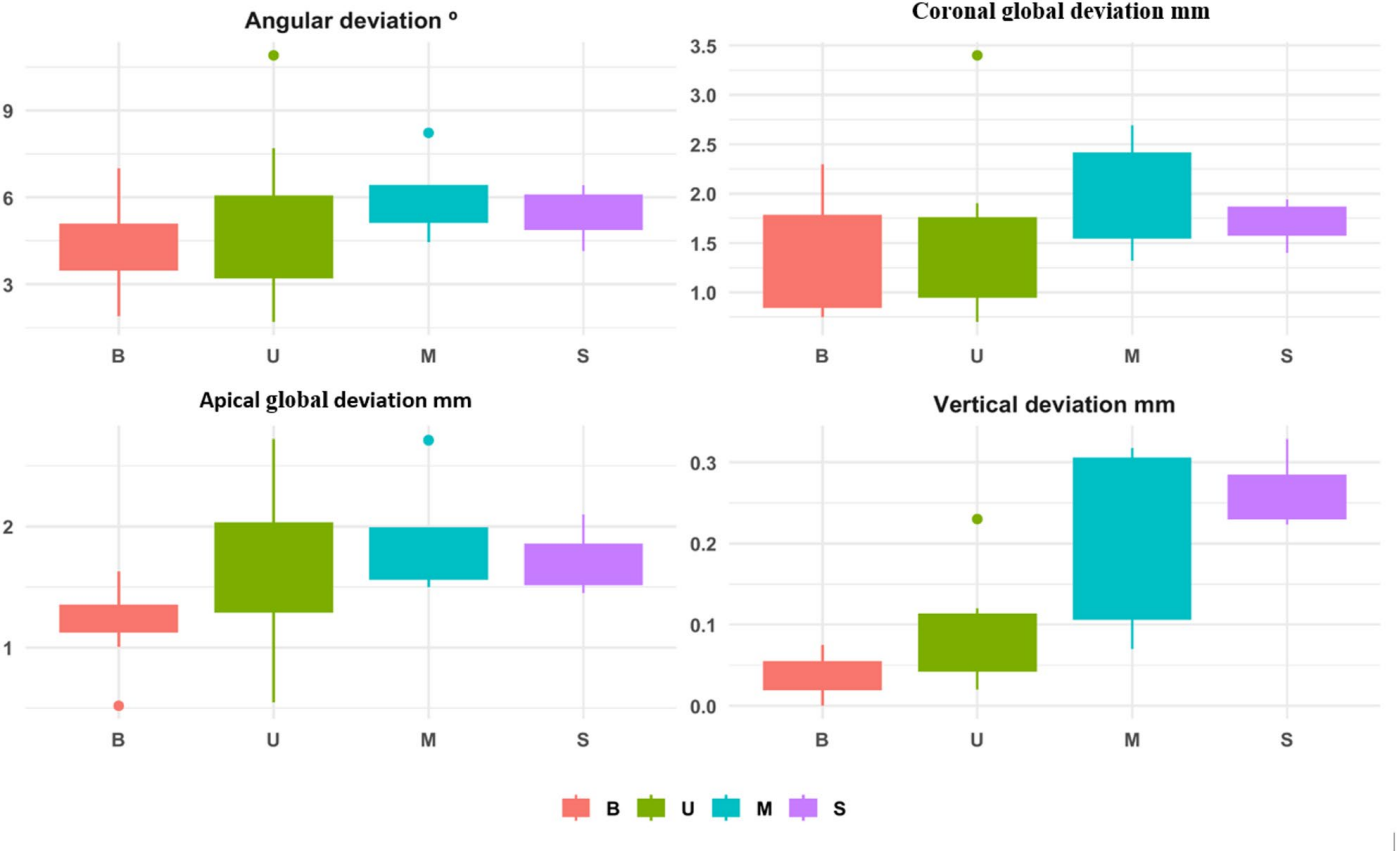
Box-plots presenting coronal global and apical global deviations, as well as vertical discrepancies of the four types of fully guided surgical guides

For bilateral tooth-supported guides, coronal global deviation was 1.32 ± 0.54 mm, apical global deviation was 1.22 ± 0.3 mm, while deviation in the vertical direction was 0.04 ± 0.03 mm. Coronal global deviation of unilateral tooth-supported guides was 1.42 ± 0.71 mm, apical global deviation was 1.59 ± 0.58 mm, and vertical deviation was 0.09 ± 0.05 mm. For mucosa-supported guides, coronal global deviation was 1.99 ± 0.62 mm, apical global deviation was 1.89 ± 0.56 mm, while deviation in the vertical direction was 0.20 ± 0.13 mm. Coronal global deviation of stackable guides was 1.70 ± 0.28 mm, apical global deviation was 1.72 ± 0.34 mm, and vertical deviation was 0.26 ± 0.06 mm (Fig. 6).

## Discussion

The primary outcome of this clinical trial was to compare angular deviation among four types of fully guided surgical guides: bilateral tooth-supported guides, unilateral tooth-supported guides, mucosa-supported complete-arch guides, and stackable guides. The analysis failed to reject the null hypothesis, as no statistically significant differences in angular deviation were detected among the four groups.

With respect to secondary outcomes, the Games–Howell post hoc test demonstrated no statistically significant differences among the groups for coronal global deviation or apical global deviation. In contrast, a statistically significant difference was observed for vertical deviation; therefore, the null hypothesis was partially rejected with respect to the secondary outcome measures.

In the context of digital implant dentistry, trueness and precision, which are two vectors for accuracy, are key concepts for assessing the performance of guided surgery systems. Trueness refers to how close the actual implant position is to the planned position. Precision, on the other hand, refers to how consistent or repeatable the guided surgery results are [4, 5]. The present study was conducted in patients (in vivo); therefore, the findings are expected to reflect real clinical conditions. Factors such as tongue and cheek interference, degree of mouth opening, patient movement, and intraoperative compliance may influence surgical guide stability and the accurate execution of the procedure. In contrast, surgical guides evaluated in cadaver-based (ex vivo) studies often demonstrate greater deviations, which may be attributed to tissue alterations occurring after specimen defrosting that may adversely affect the complete passive seating of the surgical guide [15]. In contrast, guided implant surgery performed in models (in vitro) demonstrated a high degree of accuracy, which can be attributed to improved accessibility during the procedure, straightforward verification of complete guide seating, and ease of guide fixation [3].

There are two methods of assessing the accuracy of the surgical guide used. The first one is the radiographic matching method, in which the patient was exposed to CBCT after the surgery to locate the implants exactly, and then compare the real implant locations from the DICOM files with the virtual implant locations from the planning STL. Metal implant artefacts, patient movement during acquisition, and the exact parameters of the CBCT may affect the accuracy of the final DICOM files, which in turn may affect the accuracy of the alignment [16]. In the present study, the “digital registration method” has been utilized, which avoids a second postoperative CBCT scan and overcomes the disadvantages described above [17]. This method identifies postsurgical implant positions by means of a digital impression acquired with scan bodies attached to the implants. The STL files generated from preoperative virtual planning are superimposed onto the STL data obtained from the postoperative digital scan. This digital workflow can reduce the influence of CBCT image quality on the accuracy of data registration [12, 18].

Varga et al. compared different levels of surgical guidance—full guidance, partial guidance, and pilot drill guidance—with freehand implant placement, demonstrating that increasing the degree of guidance was associated with improved accuracy, with freehand surgery showing the lowest accuracy and fully guided surgery achieving the highest accuracy. The angular deviation was 4.3° for partial guidance and 3.04° for the fully guided technique [6]. The current study reveals that the angular deviation among the four groups was greatest in the mucosa-supported guide, which exhibited the highest value (mean 5.96°±1.61), followed by the stackable guide (mean 5.42°±1.16), unilateral tooth support (mean 4.89°±2.42), and bilateral tooth support, which had the lowest angular deviation ( 4.35°±1.47). In Varga’s study, the majority of defects were single gaps, which could account for the smaller mean value of angular deviations compared to the current research [6, 19].

A recent systematic review by Floriani, which included 10 studies published between 2018 and 2023, compared angular deviation in fully guided and partially guided tooth-supported templates with free-hand surgery. The results showed that fully guided systems have less angular deviation than partially guided and freehand implant surgery in partially edentulous arches. The fully guided approaches, angular deviations ranged from − 0.32° to 4.96°, in partially guided surgical guides from 0.59° to 3.44°, and freehand approaches from 1.40° to 7.36° [20]. The authors of a previous review emphasized the influence of the initial drilling step on overall procedural accuracy, as this step largely determines the long axis of the osteotomy, which is difficult to correct if drilling is initiated in an incorrect direction. Angular deviation is particularly critical in the esthetic zone, where significant deviation from the planned axis may result in labial surface perforation by the access channel. Such situations may necessitate the use of cement-retained restorations, with their associated drawbacks, instead of screw-retained restorations. Furthermore, in full-arch rehabilitations, unfavorable angulation of one or more implants may complicate the path of insertion [6].

To the authors’ knowledge, this is the first clinical trial that compares all types of static surgical guides (ranging from bilateral tooth support to stackable bone guides) in one study. In the retrospective study by Cassetta et al., 111 implants were used with tooth-supported, mucosa-supported, and bone-supported (not stackable) surgical guides. The tooth-supported guides showed the highest accuracy (1.10 coronal deviation, 1.36 apical deviation,3,35° angular deviation), followed by the bone-supported guides (1.18 coronal deviation, 1.62 apical deviation, 5.08° angular deviation) and mucosa guides (1.63 coronal deviation,2.10 apical deviation, 4.71° angular deviation). Paired comparisons revealed that bone-supported guides exhibited superior accuracy compared to mucosa-supported guidance in terms of apical (*P* = .030) and coronal (*P* = .004) deviations. Tooth-supported guidance exhibited superior accuracy relative to mucosa-supported guides for apical (*P* = .013) and coronal (*P* = .014) deviations. The authors emphasize the importance of fixation screws in improving all parameters, but showed statistically significant better results for only angular deviations (*P* = .006) [21].

The current study showed no statistically significant difference regarding angular, coronal global and apical global deviation. In contrast, for vertical discrepancies, there was a statistically significant difference among the four groups (*P* < .001). Thus, with the available data, the null hypothesis was partially rejected. For the vertical deviation, a significant difference was found between the four groups, with the bilateral tooth-supported guides revealing the least vertical discrepancy (0.04 ± 0.03 mm). In contrast, the stackable guides were associated with the highest vertical deviation (0.26 ± 0.06 mm). These findings are to some extent in line with the results reported from a recent case series study regarding the accuracy of stackable guides. The mean deviation between the planned and actual implant positions was 0.87 mm (± 0.50 mm) at the coronal level and 2.04 mm (± 0.69 mm) at the apical level. The mean angular deviation was 2.67◦ (± 1.25◦), which is far below our study (mean 5.42°±1.16). This may be attributed to the attachment system between the guides, in which the case series by Cristache utilized a special matrix patrix system; our study used conventional magnets between the guides [11].

A recent systematic review comparing tooth-supported guides (unilateral and bilateral) to mucosa-supported guides revealed that bilateral tooth-supported guides range within 0.46–1.47 mm in global coronal deviation, 0.28–1.77 mm in global apical deviation, 1.4–4.74° in angular deviation, and 0.03–0.84 mm in vertical deviation. Global coronal deviations of unilateral tooth-supported guides are between 0.21 and 1.2 mm, global apical deviations between 0.67 and 1.45 mm (in vivo), and angular deviations between 3.1 and 5.62 mm. Global coronal deviations of mucosa-supported guides are between 0.98 and 1.987 mm, global apical deviations between 1.18 and 2.124 mm, angular deviations between 3.12–7.177° (in vivo), and vertical deviations between 0.22 and 0.64 mm. The authors concluded that bilateral tooth-supported guides exhibited similar in vivo accuracy to unilateral tooth-supported guides, and mucosa-supported guides exhibited the lowest in vivo accuracy, while there in vitro data showed low credibility due to the mechanical complexity of living mucosa tissue [8].

The limitations of the present study include the use of a single implant system with its corresponding guided surgery kit, as well as the exclusive use of one attachment system (magnets) in the stackable guide group. In addition, full-arch scanning may yield different accuracy values compared with single-tooth or partially edentulous cases, where adjacent teeth serve as stable reference landmarks and facilitate continuous image stitching [14]. A correlation between the type of arch (maxilla or mandible) and the trueness of fully guided surgery was not addressed in this study. The anatomical differences between the arches, such as access, visibility, guide support, and soft tissue characteristics, may influence the trueness of guided surgery. Future research should investigate the potential impact of arch location on the trueness and precision of guided implant placement. The postoperative STL files were superimposed onto the initial planning STL files to allow comparison between the planned and actual implant positions. Furthermore, the lack of randomization and asymmetry in guide stabilization represent additional limitations of this study.

## Conclusions

Given the limitations of this study, the trueness of the four categories of fully guided surgical guides falls within an acceptable range. Nevertheless, when utilizing mucosa-supported and stackable guides, operators should consider inherent deviations and exercise heightened vigilance.

## Data Availability

The data that support the findings of this study are available from the corresponding author upon reasonable request.

## References

[1] Al Yafi F, Camenisch B, Al-Sabbagh M. Is Digital Guided Implant Surgery Accurate and Reliable? Dent Clin North Am. 2019;63(3):381–97. 31097133 10.1016/j.cden.2019.02.006

[2] Sunil Khanna DS, Munde DBS, Baisane DPM, Shujaulla DS, Tabasum DST, Shammas DM. Surgical Guides in Implants: A Review. Saudi J Oral Dent Res. 2020;5(9):425–30.

[3] Tahmaseb A, Wismeijer D, Coucke W, Derksen W. Computer Technology Applications in Surgical Implant Dentistry: A Systematic Review. Int J Oral Maxillofac Implants. 2014;29(Supplement):25–42. 24660188 10.11607/jomi.2014suppl.g1.2

[4] Jorba-García A, Figueiredo R, González-Barnadas A, Camps-Font O, Valmaseda-Castellón E. Accuracy and the role of experience in dynamic computer guided dental implant surgery: An in-vitro study. Med Oral Patol Oral y Cir Bucal. 2019;24(1):e76–83. 10.4317/medoral.22785 PMC6344002 30573712

[5] Tahmaseb A, Wu V, Wismeijer D, Coucke W, Evans C. The accuracy of static computer-aided implant surgery: a systematic review and meta-analysis. Clin Oral Implants Res. 2018;29 Suppl 16:416–35. 10.1111/clr.13346. 10.1111/clr.13346 30328191

[6] Varga E, Antal M, Major L, Kiscsatári R, Braunitzer G, Piffkó J. Guidance means accuracy: A randomized clinical trial on freehand versus guided dental implantation. Clin Oral Implants Res. 2020;31(5):417–30. 31958166 10.1111/clr.13578

[7] Putra RH, Yoda N, Astuti ER, Sasaki K. The accuracy of implant placement with computer-guided surgery in partially edentulous patients and possible influencing factors: A systematic review and meta-analysis. J Prosthodont Res. 2022;66(1):29–39. 33504723 10.2186/jpr.JPR_D_20_00184

[8] Shi Y, Wang J, Ma C, Shen J, Dong X, Lin D. A systematic review of the accuracy of digital surgical guides for dental implantation. Int J Implant Dent. 2023;9(1):38. 10.1186/s40729-023-00507-w. 10.1186/s40729-023-00507-w PMC10597938 37875645

[9] Yang JW, Liu Q, Yue ZG, Hou JX, Afrashtehfar KI. Digital Workflow for Full-Arch Immediate Implant Placement Using a Stackable Surgical Guide Fabricated Using SLM Technology. J Prosthodont. 2021;30(8):645–50. 33938077 10.1111/jopr.13375

[10] Lan R, Marteau C, Mense C, Silvestri F. Current knowledge about stackable guides: a scoping review. Int J Implant Dent. 2024;10(1):28. 10.1186/s40729-024-00547-w. 10.1186/s40729-024-00547-w PMC11143148 38819752

[11] Cristache CM, Burlacu Vatamanu OE, Butnarasu CC, Mihut T, Sgiea ED. Predictable full digital workflow using stackable surgical templates for complete dental arch rehabilitation with implant-supported fixed restorations-case series and proof of concept. Dent J (Basel). 2024;12(11):347. 10.3390/dj12110347. 10.3390/dj12110347 PMC11593087 39590397

[12] Jacobs R, Salmon B, Codari M, Hassan B, Bornstein MM. Cone beam computed tomography in implant dentistry: recommendations for clinical use. BMC Oral Health. 2018;18(1):88. 10.1186/s12903-018-0523-5. 10.1186/s12903-018-0523-5 PMC5952365 29764458

[13] Altalla H, Alhelou H, Karaduman F, Alawawda O, Bayindir F. Comparative accuracy of photogrammetry and intraoral scanners in recordings for complete arch implant-supported prostheses: a systematic review and meta-analysis. J Prosthet Dent. 2026;135(5):e60–e70. 10.1016/j.prosdent.2025.10.059. 10.1016/j.prosdent.2025.10.059 41241558

[14] Eldabe AK, Adel-Khattab D, Botros KH. Accuracy of intraoral photogrammetry in complete arch digital implant scanning: an in vivo prospective comparative study. J Prosthet Dent. 2026;135(2):342–51. 10.1016/j.prosdent.2025.03.041. 10.1016/j.prosdent.2025.03.041 40253232

[15] Marlière DAA, Demétrio MS, Picinini LS, De Oliveira RG, Chaves Netto HDDM. Accuracy of computer-guided surgery for dental implant placement in fully edentulous patients: A systematic review. Eur J Dent. 2018;12(1):153–60. 29657542 10.4103/ejd.ejd_249_17 PMC5883470

[16] Yi C, Li S, Wen A, Wang Y, Zhao Y, Zhang Y. Digital versus radiographic accuracy evaluation of guided implant surgery: an in vitro study. BMC Oral Health. 2022;22(1):540. 10.1186/s12903-022-02585-5. 10.1186/s12903-022-02585-5 PMC9694847 36424579

[17] Marquez Bautista N, Meniz-García C, López-Carriches C, Sánchez-Labrador L, Cortés-Bretón Brinkmann J, Madrigal Martínez-Pereda C. Accuracy of Different Systems of Guided Implant Surgery and Methods for Quantification: A Systematic Review. Appl Sci (Switzerland). 2024;14:1–15.

[18] Cristache CM, Gurbanescu S. Accuracy evaluation of a stereolithographic surgical template for dental implant insertion using 3D superimposition protocol. Int J Dent. 2017;2017:4292081. 10.1155/2017/4292081. 10.1155/2017/4292081 PMC5438863 28555157

[19] Kasradze D, Kubilius R. Influence of guide support on the accuracy of static Computer-Assisted Implant Surgery (sCAIS) in partially edentulous cases using a keyless guiding system: an in vitro study. BMC Oral Health. 2025;25(1):563. 10.1186/s12903-025-05955-x. 10.1186/s12903-025-05955-x PMC11995644 40223057

[20] Floriani F, Jurado CA, Cabrera AJ, Duarte W, Porto TS, Afrashtehfar KI. Depth distortion and angular deviation of a fully guided tooth-supported static surgical guide in a partially edentulous patient: A systematic review and meta-analysis. J Prosthodont. 2024;33:10–24. 38992883 10.1111/jopr.13893

[21] Cassetta M, Stefanelli LV, Giansanti M, Di Mambro A, Calasso S. Accuracy of a Computer-Aided Implant Surgical Technique. Int J Periodontics Restor Dent. 2013;33(3):317–25. 10.11607/prd.1019 23593625

